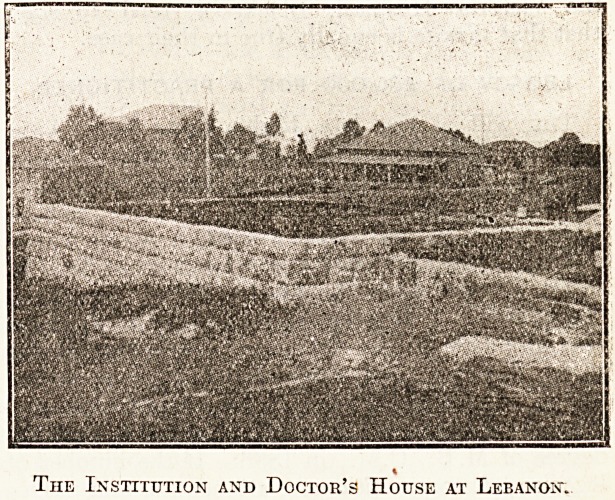# Hospital and Institutional News

**Published:** 1914-06-13

**Authors:** 


### HOSPITAL AND INSTITUTIONAL NEWS.

### THE NEW SUPERINTENDENT AT NORTH EVINGTON INFIRMARY.

A meeting of the Leicester Board of Guardians was held on May 28 to appoint a resident medical superintendent of the North Evington Infirmary and medical officer of the workhouse. Twenty-eight applications were received, and eight candidates were selected from this list and were interviewed by the Guardians. Of the selected candidates Dr. Ernest C. Hadley, M.D., B.S. Lond., F.R.C.S. Eng., resident surgeon of the Birmingham General Dispensary, was elected medical superintendent. Dr. Hadley has had considerable experience in medical and surgical work. He was house surgeon at the General Hospital, Birmingham, from October 1900 to April 1901, and afterwards resident medical officer of the Guest Hospital, Dudley; senior house surgeon at the Staffordshire General Infirmary; visiting surgeon to the hospital of the Ebbw Vale Colliery Company; house surgeon and house physician at the Birmingham Hospital for Sick Children; and resident medical officer to the Jaffray Hospital, Erdington. He has been engaged at the General Dispensary, Birmingham, as resident surgeon for many years. Dr. Hadley comes of a medical family, his father and uncle being well-known practitioners in Birmingham, but it will be noticed that the appointments Dr. Hadley has so far held have not given him large opportunities to exhibit his ability as an administrator.

### THE QUALITIES NEEDED IN AN INFIRMARY SUPERINTENDENT.

What the Leicester Guardians and the North Evington Infirmary require is a gentleman of great administrative capacity who must go straight ahead and not assume an attitude of being injured should the advice and recommendations he may submit not be immediately followed with continuous expressions of recognition of his zeal and ability. As a matter of fact the best infirmaries have always been the creation of men who, in the face of misunderstanding and opposition on the part of Boards of Guardians who have not realised the modern hospital standard, have been tireless in bringing forward again and again the improvements which their experience shows them to be necessary. In the end the often-urged request is granted, but tireless pertinacity and persistent good temper are required. The new superintendent at North Evington to succeed must have driving force and be free from all such pettiness in the discharge of his duties, which will necessarily be both onerous and difficult, especially during the first six months. Dr. Hadley comes of an able family. One of his sisters, a trained and certificated nurse, is at present matron of the Orthopædic Hospital, Birmingham, and has given proof that she does better and better work the more independent and responsible the post which she has to occupy. We hope Dr. Hadley's work in the great position he is now called upon to fill will soon afford proof that he possesses at least the high qualifications for administrative responsibility that his sister has exhibited in Birmingham. What North Evington needs in its medical superintendent is not self-consciousness, or "the injured attitude," but hard, steady, faithful work until its reorganisation is perfected in every department.

### NEW TREASURERS OF ST. GEORGE'S HOSPITAL.

Mr. F. J. Frankau did good service to St. George's Hospital by accepting the office of sub-treasurer. He has settled down to steady work, and as one result we may point to the happy selection of Lord Greville and Sir Gerard Lowther, G.C.M.G., as joint treasurers of St. George's Hospital. These gentlemen have accepted the invitation of the management of St. George's Hospital to become the joint-treasurers, and their names will be submitted for confirmation to the Court of Governors to be held on June 11 (after we go to press). Lord Greville made a most excellent chairman at the February Special Court, and as both gentlemen are men of experience, high standing, and ability we hope they will zealously enter upon their duties, and jointly with Mr. Frankau bring speedily into excellent order the domestic affairs of this hospital. Two things are essential. The first is to secure promptly that the lay governors shall have a predominant voice in the management of the hospital through its house committee; the second that steps shall be taken to find the ablest and most experienced man available for the post of financial secretary, so that the diminution in the funds of St. George's exhibited in recent years may be stopped. Such an appointment should give movement and force at least adequate to meet the ever-increasing needs of this important institution.

### THE LATEST METHOD IN HOSPITAL HISTORIES.

At last the hospital historian is coming into being, even annual reports contain a list of dates nowadays, and a hospital without a published history is like a peer without an heir—namely, threatened with extinction for lack of advertisement. We have been very interested, therefore, to receive a handsome volume consisting of 150 pages packed with illustrations, descriptive writing, and many evident indications of historical research, entitled "Sixty-Five Years' Work: Seen from Within and Without," which is described on the title-page as a "Historical Sketch of the London Homœopathic Hospital," which is published for distribution at the institution's buildings, Great Ormond Street and Queen Square. In a brief foreword Mr. Edward A. Attwood, the secretary, explains that "in these days even hospital managers cannot afford to disdain the arts of bold advertisement," and that the pamphlet has been compiled to serve as an incentive to public generosity. His use of the word "even" indicates how lately hospital managers have come to realise the importance of advertisement in any but the hackneyed and largely played-out methods of the past generation; but when we observe the interest of and the brains put into this latest miniature hospital history we see that hospital officers will never draw the moral from their own work in assisting to raise the standard of present-day social conditions (on which Mr. Attwood properly insists) unless they also see that hospitals, by freely entering into modern advertising, may rescue that too from blatancy or mere vulgarity, because they are in the unfortunately rare position of having something of genuine public importance to "announce." This brochure is a case in point.

### THE HOMŒOPATHIC HOSPITAL'S HISTORY.

Packed with capitally chosen illustrations, it comprises a bird's-eye view of the several London Homœopathic Hospitals in Golden Square and Great Ormond Street, with accounts of the successive developments at the latter place; notices of homœopathic hospitals throughout the country; a sketch of Hahnemann and his work, with the development of his theory, including the change in the public respect for it, as seen, for instance, in a famous *Punch* cartoon which is reproduced; a note on homœopathy, an account of the present hospital's work, an invitation to pay it a visit, an appeal, and last, but by no means least from an institutional point of view, some thirty-five pages of advertisements, which we are told have met the cost of publication. Only one criticism of the workmanship and editing of this history suggests itself, and that is the absence of a contents and an index. Almost every page is illustrated, and as all are full of allusions the absence of these two desirable features in a volume of any pretension is a real defect.

### THE SECRET OF HOSPITAL ADVERTISING.

In describing Mr. Attwood's brochure for institutional readers we have purposely confined ourselves to a consideration of its value as an advertisement. Now it is hardly an exaggeration to say that, in spite of all the money spent and all the large, loose talk on the subject, the average hospital manager seldom puts any brains into this department of his work. If he make an effort at all it is often simply a press effort, the following of commercial devices by merely exploiting sentiment, poverty, disease, much as if he were an Italian beggar showing appalling ulcers to English tourists on the steps of a church. What is wanted is not imitation of commercial advertisements, but initiation on institutional lines. As hospitals are not profit-hunting concerns, but really depend for their income on the value of the public work which they do, they can afford to better commercial methods even in advertisements. Any of our readers who happened to study our Hospital Sunday Number last week will have observed that every contributor from the Chief Rabbi to the leader-writer was not beating a hollow drum when insisting on the religious inspiration which, as a fact, does come to those people who are engaged in personal service and institutional work. There was the unmistakable ring of sincerity in their words. Now, unless a hospital manager really shows this sincerity, and believes, in short, that while he must be paid a salary commensurate with his work, he is yet but the trustee of his own life for public purposes (which used to be called doing the will of God), he will never make a first-rate administrator, nor be able to grasp the secret of hospital advertising.

### THE HOSPITAL STANDARD IN ADVERTISING.

Such questions as the value of motor-omnibus advertisements; how far the "appeals" in the daily press earn their cost in keeping open the columns of the newspaper to letters on the hospital's behalf, he will, of course, have to master. But these are details. The central fact is to make them share his own enthusiasm—if, as we say, he really has it—by interesting the public in hospital work. This means to encourage visiting, to found a variety of subsidiary organisations, to make his hospital the centre of a circle of instructed enthusiasm. To retain the attention of the public he must not be content to catch it—by a new cry or an old story. He must *arrest* it, and the only way to do that is to keep the public and his immediate supporters fully informed of the progress of the work and the latest requirements. Annual reports, dry statistics, balance-sheets, by themselves are not sufficient. That is why we welcome Mr. Attwood's historical Sketch of the London Homœopathic Hospital, and hope that it is the beginning of a leaven which will even improve trade advertising in the same way as the hospital spirit has improved social conditions in other respects. The catchpenny method may get a donation: it will never increase subscriptions. Perhaps the difficulty of securing these is a reflection on the faulty advertising of the modern hospital, managers of which should study the methods employed in the production of this book.

### THE BLACKPOOL HOSPITAL DEADLOCK.

It would have been an unprofitable and, administratively speaking, a degrading proceeding to have recorded the continual recrimination, and signs of want of any sense of responsibility, which still make the Victoria Hospital, Blackpool, the laughing-stock of the hospital world. Every week almost nominal changes which effect nothing, and appointments that either have to be altered or yet have the effect of exacerbating the dispute, are made, unmade, or modified. Suffice it to say that a mediation committee has been appointed, so has another house surgeon, Dr. Atkinson-Fleming, the latter with the object apparently of rendering the continued boycott on the part of the Blackpool profession as little innocuous as it is found to be. The local profession have refused to give way in the demand for the resignation of Dr. Molloy's seat on the board, but have been more successful, we understand, in their demand for representation. Dr. Molloy, whose term of office expires in March 1915, has determined not to accede to the wishes of his brother practitioners, and therefore if the present sense of all administrative irresponsibility continues to possess the board and the hospital's subscribers—who, as we previously noted, could have demanded a constitutional and business-like settlement of the whole dispute long ago had they had the public spirit to insist upon it—the deadlock may continue till the expiration of Dr. Molloy's term. The new matron, Miss Borton, entered on her duties this month. Only one point need be emphasised—namely, that a body of men who have succeeded in alienating the whole medical profession in their locality are clearly unfit for administrative responsibility, and though no experienced person supposes all the fault to be on their side, the onus rests with them to put an end to an intolerable impasse, for the existence of which they must be held responsible. However, a public of subscribers, as all experience proves, get the hospital which they deserve; and up to date the inertia, want of grip, and absence of public spirit in the subscribers to the Victoria Hospital, Blackpool, tend to show that that maxim is equally true in their case.

### LEGACY OF £30,000 FOR A PRACTITIONER.

The will of the late Right Hon. Lady Anna Caroline Chandos Pole, of Kensington Palace Gardens, who died in April at the age of eighty, contains a remarkable legacy to her medical attendant. She left estate valued in round figures at £140,000 net personalty, and bequeathed £30,000 to Dr. Thomas Ross Macdonald, M.B., of Cadogan Place, S.W., "in recognition of his great kindness and attention to me for many years past during many illnesses, and especially during my severe attack at Vittel, Vosges." Dr. Macdonald is a graduate of Glasgow University, at which he took the degree of M.B., C.M., in 1886. If the number of legacies left to practitioners, attendants, and nurses be relatively small, from time to time cases occur like the present, in which a wealthy patient bequeaths a small fortune to her medical man or nurse. Possibly these cases are becoming rarer owing to the fact that the general practitioner in these days occupies, on the whole, a less intimate position in the affairs and affections of his patients than the family doctor did a generation ago. The term "family doctor," indeed, has an obsolete sound, and its passage into the limbo of terms which now express no reality may be seen in its capture by commercial firms to designate volumes intended for the more credulous, less intelligent, and uneducated public. People change their doctors more than they used to, quick consultations, like quick lunches, are fashionable, and it is to be observed that the testator in this case belonged to a generation whose habits of mind have for the most part passed away.

### THE HOSPITAL SUNDAY CRISIS.

June 14 is Hospital Sunday in London, and upon the results of the collections upon that day this year may depend the continuance or discontinuance of this great Fund in the near future. The huge falling-off in the yield at the churches and chapels, a correspondent suggests, is due to the injurious effect of the Insurance Act. We are confident that no intelligent person of either sex will withhold their contribution on Hospital Sunday because trivial cases are not now treated as out-patients and employers have to pay insurance premiums. Is it not a diminution in the faith in and work for the hospitals of not a few religious teachers and ministers which has crippled Hospital Sunday in recent years? A few of the clergy work zealously still, but the majority, judged by the small sums they collect, are lukewarm, if they are not even something less. We are convinced that if the neutral platform for all the churches afforded by Hospital Sunday is destroyed, much of the influence exercised by the clergy and ministers with the people must necessarily be destroyed with it. Wake up, clergy, ministers, and congregations! Realise how much more in benefit the hospitals of London are giving to your poor people to-day than ever before. Make Hospital Sunday this year by a special effort produce an adequate recognition of the splendid aid the hospitals render to the churches, congregations and ministers every day throughout the year.

### GERMAN NATIONAL INSURANCE INSTITUTIONAL STATISTICS.

In Germany they treat in institutions, under a national insurance scheme, not only, as with us, tuberculosis, but also alcoholism, sexual diseases, a few maladies such as rheumatism, and, since 1912, dental affections. This being so, it is not surprising to learn that the cost is now considerable, having grown, on the authority of a publication issued by the German National Insurance Office, from two million marks in 1897 to twenty-seven million in 1911, and to thirty million in 1912. The insurance institutions numbered 83, of which 41 were tuberculosis sanatoriums. More of the latter are being built. Of forest camps great use was made, and this is not surprising, Teutonic sensibility to natural scenery, and especially to the beloved *Tannenbaum*, being so great. The tuberculosis results were about what has now come to be recognised as the usual—namely, good in nearly 50 per cent., as estimated after the lapse of five years. It is stated, however, that these are tending to improve year by year, and, which is also encouraging, early cases are now coming for treatment in greater numbers. In the important matter of relapse after treatment, men seem to suffer more than women, no doubt from having to go back to breadwinning earlier. Our Insurance Committees should note that this report does not speak of "cure," but of restoration of working capacity.

### THE HOSPITAL LEAVEN IN PRISON CELLS.

At an inquest on a woman prisoner who had died at Holloway Prison, the Coroner, Dr. Waldo, elicited from the witnesses evidence which tended to show the state of that prison at the present time. He was informed that prisoners did not have to sleep on bare plank beds, that insect life was entirely absent from the cells, and, by Dr. Forward, that the diet was far preferable to workhouse fare. The conduct and sympathetic behaviour of the wardresses were also insisted on. In addressing the jury, the Coroner said that the evidence of the woman superintendent of the hospitals, of the wardresses, of the senior medical officer, and of the deputy governor, Mr. Forward, F.R.C.S., agreed with what he had seen for himself before the opening of the inquest, and which the jury might inspect for themselves. It pointed to clean cells, ventilated by open windows and ventilators, and heated in cold weather by hot-water pipes. The use of spring mattresses in the hospital cells and of mattresses throughout the gaol, the absence of water-closets in all the cells, the temporary use of commodes in the cells where necessary, and the position of sanitary and lavatory accommodation on the same floor as, but removed from, the cells were also remarked on by him. Each cell, he added, has an electric bell to summon wardresses day or night, and the food was well served, well cooked, and abundant—in fact, better than was usually found in workhouses.

### EFFECTS OF THE HOSPITAL STANDARD.

The Coroner also stated that at a previous inquest held by him at Holloway the evidence disclosed the fact that prisoners frequently fed sparrows and gaol pigeons with surplus bread through their cell windows. In the hospital cells the windows opened completely inwards in two halves. There can be little doubt that the progress in hygiene and administration here disclosed is due ultimately to the teaching of the voluntary hospitals. They set the example and raised the standard first among all institutions, with the result that one after another Poor-Law infirmaries, mental hospitals, and prisons were levelled up to aim at similar ideals of cleanliness, hygiene, and administration. You cannot have hospital cells in a prison up to a certain high standard without seeing its effect upon the standard of the cells as a whole. The above is a capital example of the voluntary hospital leaven in prison life.

### LONG HOURS AND SHORT HOLIDAYS.

Different speakers at a recent meeting of the visiting committee of the Croydon Mental Hospital brought both these counts against the conditions of service of different sections of the institution's staff. Councillor Thompson was followed by other speakers in pointing out that for the day attendants and nurses to be on duty from 6 a.m. to 7.45 p.m. was very excessive duty; while he also remarked that the artisans had only ten days' annual leave, which included five public holidays. He recommended that, if there were no alternative, the members of the day staff should be increased and that the artisans should have a clear week or ten days' annual leave exclusive of the public holidays. The discussion, however, seemed fruitless. The motion for the reception of the report concluded the proceedings. In days when the posts of attendant, nurse, and house surgeon are becoming difficult to fill no institution can lag behind the general improvement of the service, and where the staff conditions advance those of the artisans must eventually follow. To draw an analogy, already we have seen institutions in which besides a nurses' home, what is virtually a home for ward-maids has been added, not necessarily in a separate building, but as a department to itself. What are the views of Dr. Pasmore, the medical superintendent, on the above proposals?

### LEBANON HOSPITAL FOR MENTAL CASES.

Since the opening of the Lebanon Hospital for mental cases, near Beyruth, Syria, in 1900, some 1,264 patients have been received of various types, for this institution is both international and undenominational in character. There has now been added a new English House, and we append an illustration of the building. As showing the wide interest taken in this mental hospital, it

The Institution and Doctor's House at Lebanon.

appears from the report of the London General Committee (on which is Dr. Sandwith, whose historical sketch of nursing prior to 1850 appeared in our columns last week), that subscriptions and donations towards its maintenance are received from six countries, apart from the United Kingdom. Mr. T. Waldmeier founded the institution in 1896.

### COMPETITIVE DESIGNS AT BURNLEY.

A week or two ago the board of management of the Burnley Victoria Hospital decided to hold a limited competition for architects in reference to the hospital extension. The assessor in the competition was Mr. H. Burton Adams, F.R.I.B.A., who has been instructed to examine the plans submitted and to report to the committee. Of the fifty-seven applications to compete the names of twelve architects were ultimately selected. These were Messrs. John Brooke and Elcock, Manchester; Mr. Hugh Healey, Manchester; Mr. Arthur Marshall, Nottingham; Mr. William A. Pite, London; Mr. Thos. A. Pole, London; Mr. Alfred Saxon Snell, London; Messrs. Taylor and Siminster, Oldham; Mr. Arthur W. Worrall, Wolverhampton; Messrs. Hitchon and Pickup, Burnley; Messrs. G. and S. Keighley, Burnley; Mr.

W. A. Quarmby, Burnley; and Mr. T. H. Vowles, Burnley. It is a pity, as we have often remarked, that hospital competitions for architectural purposes are not scrutinised more carefully than is at present the case. The past has shown that the whole standard, if the best results in the hospital sense are to be secured, needs revising, and one of the first essentials in getting this revision is institutional interest in every competition that is held. Hitherto few hospitals have felt their responsibility in this matter. They have been ready to blame the architect if they were dissatisfied with the result, and, no less disastrously, sometimes to follow him into all sorts of expensive fashions, or to yield without due consideration to the fancies of individual members of the honorary medical staff, who, however skilful as operators, have quite often proved childishly ignorant of the essentials of efficient administration, to which, of course, structural design and sometimes detail must be made to serve. The tendency has been to throw responsibility on any convenient shoulder, and not to realise that for a successful design there is needed a carefully chosen assessor, carefully drafted specifications as to what is required and how much money is available, and finally the close co-operation of the architect with the hospital administrator. Who is the expert administrator at Burnley?

### QUEEN ALEXANDRA AND ANCOATS HOSPITAL.

Queen Alexandra has consented to become patroness of Ancoats Hospital, in consideration of the associations of the late Duke of Clarence therewith, who, on October 20, 1888, laid the foundation-stone of the wing that by special permission is called the Albert Victor Pavilion. This honour has given the committee of management much satisfaction, especially in view of the fact that they are about to make a public appeal for the building of an additional ward and accommodation for the extra staff this will entail. Sir Frederick Cawley, Bart., M.P., has generously offered to start the fund with a donation of £500.

### A NEW CHILDREN'S HOSPITAL FOR DUNDEE.

We alluded only last week to the burning by suffragettes of the new women's hospital in Dundee, and in the same paragraph to the decision of the Dundee Royal Infirmary to allot a second ward of thirty cots for children as a temporary measure, since it was felt that the cost of providing a separate children's institution would be prohibitive. There is something like poetic justice, therefore, in the almost simultaneous announcement that Mr. F. B. Sharp has offered £10,000, on acceptable conditions, towards the building and endowment of a hospital for sick children. The conditions, which have been accepted by the board of the Royal Infirmary, are that the proposed institution should be managed by the infirmary's directors, and that the offer shall be withdrawn unless the necessary funds for building and equipment be forthcoming within two years. The Lord Provost's Public Committee, which was appointed at a meeting recently called by him, will probably take charge of the necessary appeal.

### THE MISSION HOSPITAL PROBLEM.

The remarks which we made last week in connection with medical missions, occasioned by the celebration which takes place at Livingstone College to-day (Saturday), are illustrated by some interesting criticisms made of late by Mr. C. J. Bond, F.R.C.S., at Leicester. In dealing with medical missions, he said that there were two dangers in this branch of work. First, he said, there was the danger lest the proper standard of medical efficiency should be lost sight of; and, secondly, that the surgical aspect should engross attention from the educational side of the work. He urged, therefore, that what was wanted was the establishment of hospitals, which he described as among the great reforming agencies of the world. He alluded also to the place of women in the medical mission field, and said that there was much scope for them in the Zenana Mission. We observe that at to-day's ceremony at Livingstone College a member of the Mengo Mission Hospital in Uganda is among the speakers, and he will, no doubt, not only insist on the value of elementary medical training in the mission field, but also will describe exactly how the mission hospital has learnt to deal with the particular problems on which Mr. Bond recently laid stress.

### DEVIZES TOWN HALL AS A HOSPITAL.

On Bank Holiday the Town Hall at Devizes was taken possession of by various local voluntary aid detachments of the British Red Cross Society and converted into a "hospital with sixteen beds" and provision for eight sitting-up patients. An "operating theatre" was also set up, as well as kitchen, nurses' room, sterilisers, etc. The local Corn Exchange was also used as a hospital with two wards with "theatre" and "pharmacist's stores," while wagons and vans were fitted up with sling stretchers, etc., for the reception of "cases." Among those taking part were members of the Devizes, Calne, and Pewsey detachments, and the inspecting officer was Major Maurice, R.A.M.C. We have often commented on the fact that the voluntary aid detachments, good as they are within limits, are so much discouraged and sometimes starved by the military authorities that their efforts are often more a credit to their patriotism than to professional efficiency and organisation.

### SIR WILLIAM OSLER'S CRUSADE.

On Thursday last week the new pathological laboratory which has recently been built at the Royal Mineral Water Hospital, Bath, was opened by Sir William Osler, Regius Professor of Medicine at Oxford University. The laboratory, which is situated on the top of the detached building in the garden, has been added for research work in relation to rheumatoid arthritis and kindred diseases. Sir William Osler, who was received by the Mayor of Bath, Dr. Preston King, after making a tour of the Roman baths, bathing establishment, and mineral water hospital, declared the new laboratory open. In doing so he emphasised the need and value of laboratory work both to the institution and the individual medical man, and expressed the hope that a suitable library would be formed at the institution—a hope which he further encouraged by a donation of £10 towards its realisation. Those who remember Sir William Osler's address to the British Hospitals Association at the Oxford Conference last year will remember the stress which he laid on the value of the pay-ward and of hospital laboratories. That he is active in prosecuting his proposals this visit and speech at Bath sufficiently show.

### NEXT WEEK'S CONFERENCE AT NEWCASTLE.

On June 18—that is, Thursday next—the British Hospitals Association will hold its annual conference at Newcastle, of which the programme has been published already in these columns. Those London hospital men who are attending will probably for the most part travel up together by the 2.20 train from King's Cross. A new announcement has also to be made in relation to the Guide which has been written and prepared for the use of members. We understand that the Lord Mayor is making himself responsible for this pamphlet, which will contain not merely succinct and expert information, but also several carefully chosen illustrations; while arrangements have also been made for an expedition, conducted by those best able to show the points of interest, to the Roman Wall, Durham Castle and Cathedral, and, we believe, for a river trip. It remains only to be added that we hope that there will be a good attendance. The subjects of the papers, as our readers have learnt, are of urgent and practical importance, and wideawake officials anxious to exchange experience and add to their knowledge may be earnestly advised to attend.

### SEASONAL LUNACY AND OVERCROWDED WARDS.

A report upon the observation wards at the Lambeth Workhouse has revealed a state of overcrowding necessitating some inmates sleeping on the floors. In the case of the women's ward it was reported that it was even possible that some of the quiet ones would have to be turned out of their beds to make room for those more seriously ill. The trouble is apparently due to the seasonal tendency, for a spell of bad weather often results in many people having to be placed under observation, and as it is often not possible to get such cases certified there at times comes to be overcrowding. The Guardians, however, are taking steps to prevent such a state of affairs, but if "seasonal lunacy" is to be accepted as a cause of overcrowding it cannot also be accepted as an excuse for lack of accommodation. A regular emergency demands regular provision to meet it.

### THE DISPENSERS' HALF-HOLIDAY.

The decision of the Home Secretary that the dispensers are entitled to a weekly half-holiday under the Shops Act, referred to in a recent number of The Hospital, has been anticipated by the Camberwell Board of Guardians. Last summer they decided to close their dispensaries at two o'clock on Saturday afternoons, and made arrangements with local chemists for the dispensing of any prescriptions after that hour. The experiment proved so satisfactory and, we understand, caused such slight inconvenience that the Guardians have decided again to close the dispensaries on Saturday afternoons not only in the summer-time, but also during the winter months. Whilst the Shops Act may not apply to dispensaries in infirmaries, an arrangement similar to that arrived at in Camberwell might be made whereby the dispensers may have their half-day off.

### THE DEFICIENCY IN THE DRUG FUND.

More definite information has now become available with regard to the deficiency in the Insurance drug funds, and the number of deficiency areas is larger than most people had supposed. There are in England and Wales 139 Insurance areas, and from particulars which have been collected by the Pharmaceutical Society it is seen that there is a deficiency in the drug fund in no fewer than thirty areas, and that in forty other areas a small balance is still owing to the chemists on their accounts for the first year. For the chemists in the areas affected the position is somewhat serious, and in view of the increased demand on the drug funds during the first quarter of the present year it is obvious that unless something is done to relieve the situation chemists will be in a very unhappy position. Mr. Masterman, Chairman of the Joint Insurance Committee, has consented to receive a deputation appointed by the Council of the Pharmaceutical Society, and pharmacists are hoping that as an outcome of this interview steps will be taken to secure them from inconvenience and loss.

### THIS WEEK'S DRUG MARKET.

The tone of the market seems healthy, although business has not been on a very large scale. Cod-liver oil is still somewhat inclined towards higher prices, but it does not seem probable that any substantial advance will take place, and there does not appear to be any immediate necessity to make large purchases. Buchu leaves are dearer, and still higher prices are expected. There is no change in the position of camphor, but it might not be unwise to make purchases, as any alteration would probably be in an upward direction. Citric acid has again advanced in price, and still higher values are expected. Tartaric acid has a dearer tendency. Quotations for chloral hydrate have been raised. Chrysophanic acid has again advanced in price. Quotations for salol have been reduced. In view of the firmer tone of cocaine it might not be inadvisable to replenish stocks of this drug at the low prices now ruling. The high value of belladonna-root is well maintained, and there seems to be no immediate prospect of a decline. A fair business has been done in quinine, and an advance in price would not come as a surprise. The position of opium has undergone little change, but the tendency of prices seems to be in an upward direction.

## Figures and Tables

**Figure f1:**